# Maternal, fetal and perinatal alterations associated with obesity, overweight and gestational diabetes: an observational cohort study (PREOBE)

**DOI:** 10.1186/s12889-016-2809-3

**Published:** 2016-03-01

**Authors:** Staffan K. Berglund, Luz García-Valdés, Francisco J Torres-Espinola, Mª Teresa Segura, Cristina Martínez-Zaldívar, María J. Aguilar, Ahmad Agil, Jose A. Lorente, Jesús Florido, Carmen Padilla, Signe Altmäe, Acensión Marcos, M. Carmen López-Sabater, Cristina Campoy, on behalf of the PREOBE team

**Affiliations:** Centre of Excellence for Paediatric Research EURISTIKOS, Department of Paediatrics, School of Medicine, University of Granada, Avda. De Madrid 11, 18012 Granada, Spain; Department of Clinical Sciences, Pediatrics, Umeå University, 901 85 Umeå, Sweden; Department of Pharmacology, University of Granada, Avda. de la Investigación 11, 18016 Granada, Spain; Department of Legal Medicine, University of Granada, Avda. de la Investigación 11, 18016 Granada, Spain; Department of Obstetrics and Gynaecology, University of Granada, Avda. de la Investigación 11, 18016 Granada, Spain; Department of Paediatrics, University of Granada, Avda. de la Investigación 11, 18016 Granada, Spain; Technology and Nutrition (ICTAN), Spanish National Research Council (CSIC), Institute of Food Sciences, José Antonio Novais 10, 28040 Madrid, Spain; Department of Nutrition and Bromatology, University of Barcelona, Joan XXIII 27-31, 08028 Barcelona, Spain

**Keywords:** Pregnancy, Maternal overweight, Maternal obesity, Gestational diabetes, Offspring, Fetal nutrition, Early programming, Vitamin B12, Folate, Iron status, Glucose metabolism

## Abstract

**Background:**

Maternal overweight, obesity, and gestational diabetes (GD) have been negatively associated with offspring development. Further knowledge regarding metabolic and nutritional alterations in these mother and their offspring are warranted.

**Methods:**

In an observational cohort study we included 331 pregnant women from Granada, Spain. The mothers were categorized into four groups according to BMI and their GD status; overweight (n:56), obese (n:64), GD (n:79), and healthy normal weight controls (n:132). We assessed maternal growth and nutritional biomarkers at 24 weeks (*n* = 269), 34 weeks (*n* = 310) and at delivery (*n* = 310) and the perinatal characteristics including cord blood biomarkers.

**Results:**

Obese and GD mothers had significantly lower weight gain during pregnancy and infant birth weight, waist circumference, and placental weight were higher in the obese group, including a significantly increased prevalence of macrosomia. Except for differences in markers of glucose metabolism (glucose, HbA1c, insulin and uric acid) we found at some measures that overweight and/or obese mothers had lower levels of transferrin saturation, hemoglobin, Vitamin B12 and folate and higher levels of C-reactive protein, erythrocyte sedimentation rate, ferritin, and cortisol. GD mothers had similar differences in hemoglobin and C-reactive protein but higher levels of folate. The latter was seen also in cord blood.

**Conclusions:**

We identified several metabolic alterations in overweight, obese and GD mothers compared to controls. Together with the observed differences in infant anthropometrics, these may be important biomarkers in future research regarding the programming of health and disease in children.

**Trial registration:**

The trial was registered at clinicaltrials.gov, identifier (NCT01634464).

## Background

The concept of early programming of child health is an established and highly relevant research field [1–4]. Numerous experimental and epidemiological studies have shown that nutritional alterations in pre-, peri-, and postnatal stages of life may have a significant impact on the future health and development on a child. Associations have been found into adulthood with metabolic diseases, neurodevelopmental impairments, cancer, and immunological function alterations [2, 4, 5]. Obesity during pregnancy is one of the most established risk factors for negative long-term programming. In addition to its short term risks such as preterm birth, and macrosomia [6, 7], obesity have also been associated with increased risks of metabolic and immunological dysfunctions and of poor neurodevelopment [4, 5, 8–11]. Considering that a large proportion of women of reproductive age in developed countries enter pregnancy being overweight or obese, and the numbers are increasing, these described associations constitute a threat to future child health [12].

To better understand the intrauterine programming following maternal obesity, more knowledge is required. Obesity is closely correlated to the metabolic complication gestational diabetes (GD) and therefore, alterations in glucose regulation have been proposed as a candidate explaining the mechanism [13, 14]. It is unclear whether the previously observed correlations are applicable to non-diabetic overweight or obese pregnancies. Several other possible risk factors of non-optimal maternal metabolism or nutrition have also been proposed, such as maternal weight gain during pregnancy [15], alterations in the regulation of folate [16] or leptin [17], deficiency of vitamin D [18] or iron [19, 20], or influences of the fatty acids profiles [21, 22]. But for the most, the exact role of these biomarkers is still left to explore, since most previous research is based on retrospective epidemiological studies. There is also a lack of knowledge on how these metabolic markers actually differ between healthy mothers and those with overweight, obesity, and GD respectively.

To add knowledge to the field of early programming following maternal metabolic pathologies, we designed the PREOBE trial, with the overall objectives to establish a cohort of mother-child pairs and explore the short- and long-term effects of maternal overweight, obesity and GD compared to controls, on peri- and postnatal outcomes in the mother and her offspring. Maternal nutritional biomarkers, placental function and growth were monitored during pregnancy as well as the short- and long-term health and development of the offspring. In the present paper, we aimed to describe the methodology and baseline outcomes of the PREOBE study.

## Methods

### Study design and subjects

**Fig. 1:**
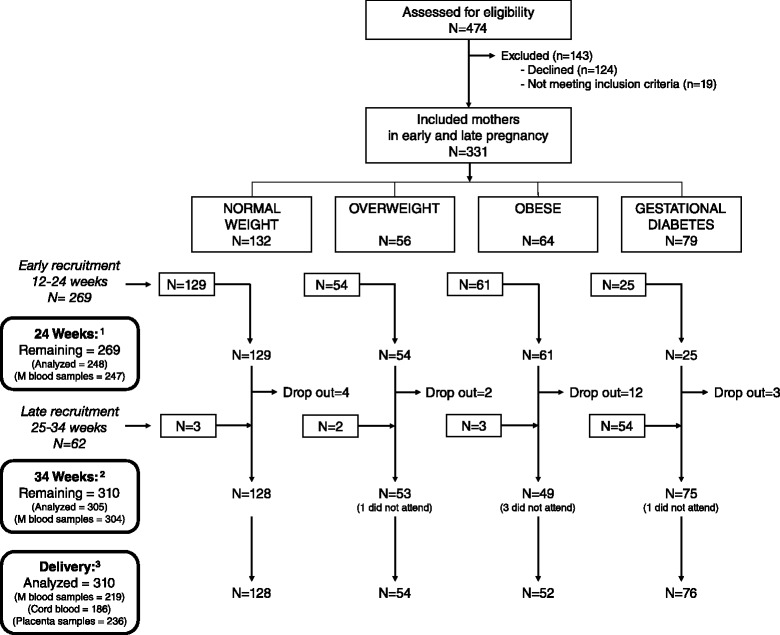
Trial profile. ^**1**^ Of the 269 mothers included before 24 weeks, 248 remained at delivery and were analyzed in the present paper including 247 blood samples. ^**2**^ At 34 weeks, 21 had dropped out and 5 cases abstained the visit, two of them due to preterm birth. Blood was drawn in 304 mothers. ^**3**^ At delivery, 245 cases called the study staff to collect blood samples from mothers (219) and umbilical cord (186), and placental samples (236). For remaining cases, only delivery record data was collected

This study was designed as a prospective observational cohort study and included no interventions. The study recruitment was performed between 2008 and 2012, through a collaboration with the Clinical University Hospital San Cecilio and the Mother-Infant University Hospital of Granada, Granada, Spain and their peripheral health centers. Pregnant women attending antenatal clinics for regular check-up were approached by study staff and invited to participate in the study. The inclusion criteria were: single pregnancy at 12–34 weeks of gestation (preferably before 20 weeks), age between 18 and 45 years, no simultaneous participation in any other research study, no drug treatment, no vegan diet, and no diagnosed diseases other than obesity, overweight or gestational diabetes. In total 474 pregnant women were assessed for eligibility. Nineteen did not meet the inclusion criteria, 15 due to pre-gestational diabetes and 4 due to underweight, and 124 did not come back for the first study visit (without stating why). The 124 cases of early drop were mostly normal weight mothers without GD (*n* = 107) but they were similar in age and educational level with those who remained. Of the 331 included mothers, 269 were included before 24 weeks of gestation. However, due to the lack of mothers diagnosed with GD at that early part of gestation, another 62 were recruited after 24 weeks (Fig. 1). All included women were allocated into four different groups based on the calculated pre-gestational body mass index (BMI) and the gestational diabetic status at 34 weeks:

1. Normal weight (18.5 ≤ BMI < 25 and no diagnosed gestational diabetes), *n* = 132
2. Overweight (25 ≤ BMI <30 and no diagnosed gestational diabetes), *n* = 56
3. Obese (BMI ≥30 and no diagnosed gestational diabetes), *n* = 64
4. Gestational diabetes (and BMI ≥18.5), *n* = 79

We actively searched for mothers in the latter three groups, and the GD group included both mothers who had already been diagnosed with GD and by those from the first three groups who developed GD between recruitment and 34 weeks and accordingly switched group. The diagnosis of GDM was made by the local clinicians at the hospital and was based on an oral glucose tolerance test (OGTT), interpreted according to the National Diabetes Data Group criteria [23] and the Third International Workshop-Conference on Gestational Diabetes Mellitus [24]. To increase the sample size, we included mothers from all three weight categories. Consequently, the GD-group included women with normal weight (*n* = 32), overweight (*n* = 23), and obesity (*n* = 24). According to the local hospital routines at time of the study, the mothers diagnosed with GD were offered to participate in an endocrine-nutritional programme to optimize glucose control using nutritional and lifestyle recommendations. In some cases they received medical treatment including oral antidiabetics or insulin therapy if they needed it. This intervention was not part of the present study and neither monitored individually. The overweight and obese mothers without GD received no similar intervention.

### Ethics, consent and permissions

The project was approved by the Bioethical Committees for Clinical Research of the Clinical University Hospital San Cecilio and the Mother-Infant University Hospital of Granada, Granada, Spain. An ethical approval was also obtained by the Research Bioethical Committee of the University of Granada. Written informed consent was obtained from all participants at study entry, and after received full information from a research group member.

### Data collection during pregnancy

The data collection procedures during pregnancy and at delivery are summarized in Table 1. At the recruitment visit, information was collected regarding maternal pre-pregnancy weight, used for calculation of the pre-pregnancy BMI, and sociodemographic and medical background including ethnicity, education, age, number of previous pregnancies/children, smoking habits, and alcohol consumption. The pregnant women were also asked about prior or current supplements of vitamins or minerals. At 24 and 34 weeks of gestation, all pregnant mothers enrolled were called back for a visit including an anthropometric revision, bio-impedance measurements, and collection of maternal venous blood, urine, cheek cell and fecal samples. The fecal samples were analyzed for microbiota as has been described elsewhere [25]. Furthermore, maternal dietary habits and physical activity were recorded by several questionnaires, and fetal growth and development was assessed by ultrasound measurements.

**Table 1:** Schedule of data collection, assessments and laboratory analyses in mothers and offspring in the PREOBE-study

Measure	Outcomes	Time of collection
Baseline recruitment information	Pre-gestational BMI, ethnicity, medical history, age, education, parity, smoking, ongoing supplements	Recruitment visit, 24 wk, and 34 wk
Maternal anthropometrics	Weight, length, circumference of abdomen, waist etc., skinfold thickness of triceps, subscapularis etc.^a^	24 wk, 34 wk
Bioimpedance	BMR^a^, impedance^a^, BMI^a^, FM/FMI^a^, FFM/FFMI^a^, body water^a^	24 wk, 34 wk
Fetal ultrasound	Fetal anthropometrics^a^	24 wk, 34 wk
Urine samples	Metabolomics^a^	24 wk, 34 wk
Fecal samples	Microbiota^b^, metabolomics^a^	24 wk, near delivery
Cheek cell sample	Microbiota^a^, genetics^ab^, fatty acids^a^	34 wk (mother), birth (infant)
Food questionnaire	Quantitative and qualitative dietary analyses^a^	24 wk, 34 wk
Placenta samples	Placental weight and circumference, Lipid profile^a^, phospholipids^a^, gene expression^a^, lipid peroxidation, pTfR^b^	Delivery
Lifestyle questionnaire	Lifestyle and physical activity^a^	24 wk
Blood samples (Mother and cord blood)	Direct analyses: ESR, glucose, lipids^a^, urea, creatinine, uric acid, bilirubin, iron status, folate, TSH, amylase, CRP, HbA1c, total protein, albumin, vit B12, insulin, cortisol, IGF1^a^, IGFBP1^a^, and lymphocyte subpopulations^a^. Frozen serum/plasma: Hepcidin^b^, TfR^b^, adiponectin^a^, resistin^a^, leptin, interleukins^a^, phospholipid profiles^a^, lipid peroxidation^a^, tocopherol^a^, retinol^a^.	24 wk, 34 wk, delivery (mother and cord blood)
Perinatal records	Gender, gestational age, Apgar, delivery mode, neonatal diagnoses/admission	–
Infant anthropometrics	Birth weight, length, circumference of head, upper-arm, chest and waist, BMI, waist/height index, heel-knee, hip-knee	Birth
Meconium	Microbiota^a^	Birth
Breast milk	Fatty acids^a^, vitamins^a^	day 1, day 7, day 30

^a^Unpublished data

^b^Published elsewhere

### Data collection at delivery

All participating mothers were instructed to contact the study center at the time of admission at the delivery ward. Whenever possible, study staff attended the delivery and measured the placental weight and circumference and collected cord blood and placental samples, immediately after cord clamping. Furthermore at delivery, a venous blood puncture was drawn from the mother. Maternal weight at delivery was collected from medical records and weight gain during pregnancy calculated. Maternal data collection also included a repeated follow up until 18 months of life including postpartum questionnaire, anthropometric and bio-impedance measures, dietary records, and in a subsample also breast milk samples.

With regard to the offspring, the following perinatal data was collected at time of delivery: infant gender, gestational age at birth, infant anthropometrics including head, chest, mid-arm, and abdominal circumference, hip-knee, knee-heal, admission to neonatal ward, Apgar score, and mode of delivery (vaginal or caesarian section). Whenever possible, the offspring’s meconium and feces at different visits within the follow-up were collected and stored for gut microbiota study. Finally at birth, cheek cells were collected and stored at − 80°. The latter have been analyzed for fatty acid profiles, and different polymorphisms in genes including FADS and ELOVs and PPRG [26].

### Laboratory analyses

The maternal blood samples from 24 weeks, 34 weeks, and delivery as well as the umbilical cord blood were analyzed for hematologic and biochemical markers at the laboratory of Clinical University Hospital “San Cecilio”, Granada, Spain including the following: Hematological parameters (Hemoglobin [Hb] and mean corpuscular volume [MCV]), erythrocyte sedimentation rate (ESR), serum biomarkers (glucose, cholesterol, creatinine, uric acid, ferritin, transferrin, transferrin saturation [TS], folate, thyroid − stimulating hormone [TSH], C-reactive protein [CRP], HbA1c, vitamin B12, insulin, and cortisol). The analyses also included fatty acids profile in plasma phospholipids. Apart from direct analyses above, we analyzed lymphocyte subpopulations and serum and plasma aliquots were also stored at −80 °C for later analyses as described in Table 1. The placental samples were analyzed for fatty acids content, lipid pro-oxidation, phospholipid profiles, and expression of placental key genes related to energy, proteins, carbohydrates, iron and fatty acids transport and metabolism. The results are partly published elsewhere [20, 27, 28].

### Statistics

All statistical analyses were performed using the SPSS statistical software package for Windows (version 22.0; IBM SPSS Inc., Chicago, IL, USA). Continuous, normally distributed variables were displayed as mean and standard deviation (SD) and explored by analysis of variance, while variables showing skew distribution were presented as median and inter quartile range (IQR) and analyzed using non-parametric rank sum tests. We used ANOVA, Kruskal-Wallis rank sum test, or Chi square test to compare the four PREOBE-groups. The comparisons were performed without confounder adjustment. In outcomes with group size in the overweight or obese group less than 20, the two groups were combined together resulting in three groups to compare. In case of significant group differences, a post hoc test was used to explore the overweight, obese and gestational diabetic groups against the normal group. The post hoc tests were adjusted for multiple comparisons by multiplying the p-value with the number intergroup comparisons (two or three). P-values < 0.05 were considered statistically significant.

## Results

From the 331 pregnant women included in the current study, 21 dropped out before the visit at 34 weeks. Another 5 of them did not attend the visit at 34 weeks (two of them due to preterm delivery) but remained in the study for postnatal follow-ups. Thus, 310 mother-child pairs were considered as remaining at delivery and included in the analyses of the present paper. The compliance to participate at each visit was generally low and furthermore, some women occasionally refused blood sampling. The number of cases visiting the study center, as well as the number of blood samples drawn is described in Fig. 1. At time of delivery, we were only able to participate for data collection in a subsample due to the fact that the mothers forgot to contact the research team at time of delivery. Cord blood sampling was further limited in numbers due to the tight time limit to perform the collection.

### Maternal characteristics and outcomes

The background and baseline characteristics of the mothers who completed the study, together with their laboratory and anthropometric outcomes are shown in Table 2. With regard to the sociodemographic background, we found that GD mothers were significantly older than the controls (multiple comparison adjusted value [adj. p] was <0.001). Furthermore, the obese group had generally lower prevalence of university education (adj. *p* <0.001). However, no significant differences were found in ethnicity, parity, smoking or alcohol intake. The average weight gain during pregnancy was significantly lower in the obese (adj. *p* <0.001) and diabetic (adj. *p* <0.001) groups. The reported intake of supplements before study entry was generally high. We found a significantly lower proportion of mothers in the obese group reporting intake of folic acid (adj. *p* = 0.015) and higher proportions of mothers in the GD group reporting intake of B12 (adj. *p* = 0.039).

**Table 2:** Baseline characteristics, anthropometrics, and laboratory measures during pregnancy for the 310 mothers who remained in the PREOBE trial at delivery

	Time	*N*	Normal weight *n = 128*	Overweight *n = 54*	Obese *n = 52*	Gestational diabetes *n = 76*	*P*
Background							
Age (y)	20 wk	310	30.9 ± 4.2	32.0 ± 4.2	29.5 ± 7.8	33.7 ± 4.6^a^	<0.001
Parity (>1)	–	306	55 (43.3 %)	24 (45.3 %)	28 (53.8 %)	35 (47.3 %)	0.637
Smoking during pregnancy	–	268	17 (14.4 %)	5 (10.2 %)	7 (15.6 %)	7 (12.3 %)	0.850
Alcohol during pregnancy	–	270	6 (5.1 %)	1 (2.0 %)	1 (2.2 %)	3 (5.3 %)	0.692
Education at university-level	–	306	68 (53.5 %)	22 (41.5 %)	11 (21.2 %)^a^	27 (36.5 %)	<0.001
Supplements during pregnancy							
Iron supplementation	–	280	46 (36.2 %)	13 (28.9 %)	10 (21.7 %)	28 (45.2 %)	0.065
Folic acid supplementation	–	260	115 (92.7 %)	38 (84.4 %)	32 (76.2 %)^a^	43 (87.8 %)	0.036
B12 supplementation	–	259	64 (51.6 %)	22 (48.9 %)	22 (52.4 %)	35 (72.8 %)^a^	0.055
Anthropometrics							
Pre-gestational BMI (kg/m2)	0 wk	310	22.0 ± 1.7	23.7 ± 1.3^a^	33.3 ± 2.8^a^	27.7 ± 6.2^a^	<0.001
Weight (kg)	0 wk	310	59.1 ± 5.6	72.3 ± 6.0^a^	87.3 ± 9.1^a^	72.5 ± 18.3^a^	<0.001
	delivery	209	72.0 ± 8.8	82.2 ± 7.8^a^	94.8 ± 11.3^a^	77.9 ± 16.1^a^	<0.001
Weight gain during pregnancy (kg)	delivery	209	12.5 ± 6.1	10.3 ± 5.2	7.2 ± 6.9^a^	6.9 ± 8.2^a^	<0.001
Iron status							
Ferritin (μg/l)	24 wk	240	18.3 (10–26)	18.3 (10–28)	22.7 (16–41)^a^	23.0 (18–30)	0.033
	34 wk	299	15.7 (9–21)	12.0 (9–19)	12.8 (9–18)	16.4 (11–29)	0.011
	delivery	199	21.0 (16–33)	19.6 (16–26)	17.4 (15–22)	22.0 (18–46)	0.005
TS (%)	24 wk	242	18.9 ± 8.6	16.6 ± 6.3	15.3 ± 5.4^a^	20.3 ± 7.7	0.009
	34 wk	300	17.1 ± 10.9	14.9 ± 7.9	12.5 ± 5.2^a^	19.0 ± 13.7	0.006
	delivery	200	17.6 ± 12.4	16.4 ± 11.9	13.9 ± 6.0	17.4 ± 8.4	0.376
Hb (g/l)	24 wk	222	12.6 ± 1.6	12.4 ± 1.7	12.1 ± 0.4	11.6 ± 0.8	0.136
	34 wk	288	11.8 ± 1.2	11.5 ± 1.5	11.6 ± 0.8	11.8 ± 1.1	0.421
	delivery	199	12.6 ± 2.0	11.4 ± 1.4^a^	11.4 ± 1.4^a^	11.6 ± 1.7^a^	<0.001
MCV (fl)	24 wk	222	88.9 ± 4.4	88.9 ± 4.7	88.0 ± 4.1	89.3 ± 4.3	0.617
	34 wk	288	87.3 ± 5.5	87.0 ± 5.4	85.4 ± 4.1	86.6 ± 5.1	0.193
	delivery	198	86.2 ± 6.9	84.6 ± 5.3	84.1 ± 5.3	87.7 ± 4.9	0.036
Vitamins							
Vitamin B12 (pg/ml)	24 wk	59	321 ± 124	262 ± 76		309 ± 136	0.156
	34 wk	87	257 ± 91	248 ± 68		307 ± 101	0.012
	delivery	59	312 ± 98	221 ± 73^a^		263 ± 90	0.037
Folate (ng/ml)^b^	24 wk	242	15.3 (11.5–19.3)	16.3 (10.5–19.7)	14.4 (8.4–19.8)	18.1 (13.4–20.0)	0.212
	34 wk	300	14.8 (10.2–18.0)	13.5 (7.4–19.2)	11.4 (6.2–16.3)^a^	18.2 (12.3–20.0)^a^	<0.001
	delivery	201	14.2 (9.1–18.3)	10.1 (6.0–17.4)	7.3 (5.0–14.6)^a^	15.1 (9.6–19.0)	0.001
Other biomarkers							
Glucose (g/l)	24 wk	242	80.5 ± 16.6	87.3 ± 16.6	84.8 ± 16.7	97.2 ± 24.4^a^	<0.001
	34 wk	301	86.7 ± 18.6	88.0 ± 17.7	89.6 ± 17.8	93.5 ± 23.5	0.119
	delivery	201	82.7 ± 28.0	86.8 ± 23.7	96.9 ± 35.3	102.2 ± 33.8^a^	0.002
Insulin (μU/ml)	24 wk	104	21.8 (6.6–39.1)	36.5 (14.1–66.2)	26.9 (16.9–54.3)	21.0 (14.1–60.8)	0.306
	34 wk	159	29.9 (12.9–51.4)	38.9 (24.3–63.3)	38.4 (22.8–71.0)	24.5 (12.5–42.1)	0.013
	delivery	110	10.0 (5.4–15.7)	14.6 (10.7–27.5)^a^		12.1 (6.7–23.0)	0.008
HbA1c (%)	24 wk	196	4.2 (4.0–4.4)	4.5 (4.2–4.9)^a^	4.8 (4.2–5.1)^a^	5.1 (4.6–5.3)^a^	<0.001
	34 wk	258	4.5 (4.3–4.8)	4.8 (4.4–5.4)^a^	5.0 (4.5–4.4)^a^	5.1 (4.7–5.5)^a^	<0.001
	delivery	180	4.6 (4.4–4.8)	5.0 (4.7–4.3)^a^	5.2 (4.5–5.5)^a^	5.2 (4.9–4.5)^a^	<0.001
Uric acid (mg/dl)	24 wk	243	3.09 ± 0.62	3.06 ± 0.75	3.53 ± 0.72^a^	3.08 ± 0.51	<0.001
	34 wk	301	3.62 ± 0.77	3.59 ± 0.91	3.76 ± 0.69	3.87 ± 1.04	0.186
	delivery	200	4.55 ± 1.08	4.64 ± 1.41	4.64 ± 1.22	4.45 ± 0.96	0.843
Creatinine (mg/dl)	24 wk	230	0.60 ± 0.14	0.59 ± 0.10	0.56 ± 0.10	0.58 ± 0.09	0.239
	34 wk	301	0.61 ± 0.11	0.71 ± 0.62	0.59 ± 0.11	0.66 ± 0.15	0.087
	delivery	201	0.64 ± 0.11	0.69 ± 0.19	0.61 ± 0.13	0.68 ± 0.17	0.096
TSH (ng/ml)	24 wk	241	1.65 (0.98–2.08)	1.37 (1.06–2.18)	1.65 (1.11–2.32)	1.56 (1.17–2.50)	0.888
	34 wk	299	1.39 (1.05–2.04)	1.34 (1.08–1.98)	1.59 (0.89–2.17)	1.39 (1.04–2.07)	0.954
	delivery	200	2.24 (1.61–3.16)	2.15 (1.66–2.88)	2.09 (1.59–3.06)	2.17 (1.43–2.92)	0.781
CRP (mg/dl)	24 wk	71	0.27 (0.16–0.34)	0.42 (0.17–0.53)^a^		0.68 (0.35–0.90)^a^	<0.001
	34 wk	121	0.79 (0.43–1.2)	0.65 (0.39–1.0)^a^		0.96 (0.66–2.5)	0.005
	delivery	89	0.56 (0.27–0.82)	0.45 (0.28–0.83)^a^		1.2 (0.52–2.9)^a^	0.042
ESR (mm)	24 wk	91	35.7 ± 16.7	47.6 ± 16.3^a^	47.7 ± 14.3^a^	49.4 ± 20.1^a^	0.020
	34 wk	136	47.9 ± 25.9	60.8 ± 12.4	55.0 ± 11.6	53.2 ± 19.0	0.118
	delivery	103	39.6 ± 22.9	54.0 ± 22.9		47.3 ± 24.0	0.060
Cortisol (μg/dl)	24 wk	104	21.0 ± 6.2	18.3 ± 5.7	17.1 ± 5.0^a^	19.5 ± 5.4	0.050
	34 wk	160	22.6 ± 6.7	23.3 ± 6.4	23.3 ± 6.6	22.6 ± 7.1	0.934
	delivery	112	57.5 ± 26.5	42.0 ± 15.2^a^		48.6 ± 18.2	0.007

Data are mean ± SD, *n* (%), or median (IQR) and *p*-values are ANOVA, Chi square test, or Kruskal Wallis rank sum test respectively

^a^Significantly different from normal group in a multiple-comparison-adjusted-post hoc test (p-value was multiplied with the number of post hoc analyses [two or three])

^b^Highest detectable folate level was 20 ng/ml

The biochemical variables differed significantly in several measures. TS, Hb, vitamin B12, folate, and cortisol were lower in the overweight or obese group at some time-points, while ferritin, CRP, ESR, uric acid, HbA1c, and insulin showed higher levels in obese and/or overweight mothers compared to controls. The GD group showed lower values of Hb but higher levels in folate, CRP, ESR, glucose, and in HbA1c. Regarding creatinine and TSH no significant differences nor trends of differences were observed.

### Perinatal outcomes and cord blood analyses

The perinatal data collected at delivery are presented in Table 3. The obese group had significantly higher placental (adj. *p* <0.001), and infant weight (adj. *p* = 0.003) but also higher placental/fetal weight ratio (adj. *p* = 0.018). The prevalence of macrosomia, defined as birth weight >4000 g was significantly higher in the obese group compared to the normal group with an odds ratio (95 % CI) of 4.6 (1.4–14.9), adj. *p* = 0.018. Also the mean infant waist circumferences (adj. *p* = 0.028) and chest circumferences (adj. *p* = 0.020) were higher in the obese group. The diabetic group had a higher prevalence of caesarian delivery (adj. *p* = 0.038) and the infants had a higher weight/height ratio (adj. *p* = 0.032). We observed no significant differences between any groups in gestational age at birth or in prevalence of preterm birth or LBW.

**Table 3:** Perinatal outcomes in pregnancies following maternal overweight, obesity, or gestational diabetes compared to controls

	*N*	Normal weight *n = 128*	Overweight *n = 54*	Obese *n = 52*	Gestational diabetes *n = 76*	*P*
Mode of delivery (vaginal)	228	95 (87.2 %)	31 (72.1 %)	26 (72.2 %)	28 (70.0 %)^a^	0.036
Placental weight (g)	227	475 ± 112	512 ± 123	571 ± 133^a^	517 ± 116	0.001
Placental /fetal-ratio	226	0.147 ± 0.03	0.158 ± 0.04	0.164 ± 0.04^a^	0.155 ± 0.03	0.030
Apgar score at 5 min	234	10 (10–10)	10 (10–10)	10 (10–10)	10 (10–10)	0.357
Gestational age at birth (wk)	287	39.4 ± 1.2	39.5 ± 1.4	39.8 ± 1.4	39.2 ± 1.5	0.169
Preterm birth (<37 wk)	287	2 (1.6 %)	1 (2.0 %)	2 (4.2 %)	1 (1.5 %)	0.740
Post term birth (>42 wk)	287	2 (1.6 %)	0 (0 %)	3 (6.3 %)	1 (1.5 %)	0.148
Infant gender (boy)	298	61 (48.8 %)	25 (48.1 %)	29 (59.2 %)	43 (59.7 %)	0.327
Infant birth weight (kg)	301	3.25 ± 0.39	3.29 ± 0.49	3.49 ± 0.51^a^	3.31 ± 0.46	0.020
Low birth weight (<2500 g)	301	5 (3.9 %)	2 (3.8 %)	0 (0 %)	2 (2.8 %)	0.567
Macrosomia (>4000 g)	301	5 (3.9 %)	6 (11.3 %)	8 (16.3 %)^a^	3 (4.2 %)	0.016
Infant birth length (cm)	236	50.4 ± 1.9	50.5 ± 1.6	51.0 ± 2.3	50.1 ± 2.4	0.192
Infant BMI (m/kg2)	236	13.0 ± 1.2	13.0 ± 1.3	13.3 ± 1.4	13.3 ± 1.6	0.464
Infant waist/height index	142	0.64 ± 0.04	0.64 ± 0.05	0.66 ± 0.05	0.67 ± 0.04^a^	0.023
Infant head circumference (cm)	200	34.4 ± 1.3	34.5 ± 1.1	34.4 ± 1.6	37.7 ± 1.4	0.606
Waist circumference (cm)	147	32.3 ± 1.9	32.4 ± 2.3	33.7 ± 2.6^a^	33.3 ± 1.9	0.014
Upper arm circumference (cm)	191	10.9 ± 1.0	11.3 ± 1.4	11.3 ± 1.2	11.3 ± 1.2	0.214
Chest circumference (cm)	161	33.0 ± 1.9	33.1 ± 2.7	34.4 ± 2.4^a^	33.5 ± 1.6	0.031
Hip-knee length (cm)	188	11.0 ± 1.1	10.9 ± 1.1	10.8 ± 1.4	10.8 ± 1.2	0.916
Knee-heal length (cm)	190	10.7 ± 1.1	10.8 ± 1.0	11.2 ± 1.1	11.2 ± 1.4	0.074
Admission to neonatal ward	270	11 (9.2 %)	0 (0 %)	2 (4.3 %)	2 (3.4 %)	0.098

Data are mean ± SD, *n* (%), or median (IQR) and *p*-values are ANOVA, Chi square test, or Kruskal Wallis rank sum test respectively

^a^Significantly different from normal group in a multiple-comparison-adjusted-post hoc test

ESR in cord blood was only measured in one infant from the normal group and for this reason we could not analyze this variable. Remaining laboratory measures from cord blood are shown in Table 4. Although the overweight and obese groups were combined, the number of analyzed cases was low, and no significant differences were observed in the overweight/obese group compared to the normal group. There was an overall group differences in Vitamin B12-levels and a trend suggesting lower levels in the overweight/obese group. However, only 3 cases were analyzed in the normal group and consequently the difference did not reach significance (adj. *p* = 0.4). Similarly, due to low numbers analyzed, there was a non-significant trend of higher glucose and HbA1c-levels in cord blood from the GD-group (adj. *p* = 0.160 and 0.074 respectively). The only significant post-hoc comparison was observed in folate which was higher in the GD-group compared to controls (adj. *p* = 0.024).

**Table 4:** Cord blood analyses

	*N*	Normal weight *n* ≤ *76*	Overweight or obese *n ≤ 62*	Gestational diabetes *n ≤ 45*	*P*
Iron status					
Ferritin (μg/l)	119	168 (103–265)	163 (115–233)	139 (88–268)	0.930
TS (%)	49	60.7 ± 2.1	55.1 ± 17.7	64.3 ± 20.0	0.254
Hb (g/l)	125	16.7 ± 1.6	17.0 ± 2.4	16.3 ± 2.1	0.359
MCV (fl)	125	107.5 ± 5.4	107.0 ± 6.5	107.9 ± 3.7	0.776
Vitamins					
Vitamin B12 (pg/ml)	45	665 ± 512	436 ± 198	739 ± 333	0.005
Folate (ng/ml)^b^	150	>20 (16.6– > 20)	19.9 (15.5– > 20)	>20 (>20– > 20)^a^	0.010
Other biomarkers					
Glucose (g/l)	156	69.7 ± 21.1	66.2 ± 22.2	77.4 ± 22.4	0.050
Insulin (μU/ml)	118	3.5 (2.2–5.8)	4.4 (2.9–7.3)	5.6 (2.0–10.3)	0.126
HbA1c (%)	34	3.2 (3.0–3.5)	3.3 (3.2–3.5)	3.7 (3.3–4.4)	0.064
Uric acid (mg/dl)	132	4.92 ± 0.90	4.95 ± 1.29	4.94 ± 1.06	0.995
Creatinine (mg/dl)	131	0.60 ± 0.18	0.63 ± 0.23	0.61 ± 0.21	0.712
TSH (ng/ml)	133	11.0 (7.3–15.3)	8.4 (6.3–14.4)	8.1 (5.5–12.2)	0.270
CRP (mg/dl)	47	0.00 (0.00–0.10)	0.01 (0.00–0.03)	0.01 (0.07–0.10)	0.308
Cortisol (μg/dl)	47	13.6 ± 4.5	13.7 ± 10.3	11.6 ± 5.7	0.678

Data are mean ± SD, *n* (%), or median (IQR) and *p*-values are ANOVA, Chi square test, or Kruskal Wallis rank sum test respectively

^a^Significantly different from normal group in a multiple-comparison-adjusted-post hoc test

^b^Highest detectable folate level was 20 ng/ml

## Discussion

In the present paper we explored maternal and perinatal alterations in pregnancies following maternal overweight, obesity and GD, all suggested risk factors of non-optimal programming of long-term child health. In agreement with our hypothesis, we observed several significant differences compared to controls. All three groups of mothers showed significant differences in levels of some of the metabolic biomarkers explored. The infants born to the obese mothers differed in their anthropometrics including higher birth—and placental weight and higher placental-neonatal weight ratio. They also had a higher risk of macrosomia, while the offspring born to women with GD showed no anthropometric differences compared to controls. The cord blood analyses were limited due to sample size and we found only that offspring born to GD mothers had higher levels of folate and a non-significant trend of higher glucose and HbA1c.

The maternal group differences observed in the present paper were mostly in accordance with previous research. The GD mothers had significantly higher age. This is a previously well described correlation [29] and may be an important confounding factor in long term studies of the offspring. Regarding maternal anthropometrics, we found slower weight gain during pregnancy in obese mothers as it has been previously described [30]. This is of clinical importance since it has been shown that maternal overweight/obesity and weight gain during pregnancy are both and independently associated with increased obesity in the offspring [15]. Considering these observations, we find it relevant to follow the present cohort and explore how the maternal age and weight gain during pregnancy correlates to later health markers of the offspring in the four groups respectively.

Higher glucose and HbA1c-levels in the mothers of the GD group were expected by definition. We also observed higher insulin and HbA1c-levels in the overweight and obese mothers, suggesting alterations also in their glucose metabolism. Interestingly, there was a trend of higher glucose and HbA1c also in the cord blood of diabetic offspring. Since insulin and HbA1c are not transferred to the fetus and maternal insulin has little effect on the placenta, the observation is most likely explained by differences in glucose transfer [14]. More specifically, it is suggested that higher glucose levels in the mother causes relatively higher levels in the fetus and consequently affect its metabolism. The relevance of these non-significant observations are unclear, however they are concurrent with the ‘in utero’ hypothesis, suggesting that an increased intrauterine exposure to glucose occurs in the fetus if the levels are altered in the mother. This hypothesis has been considered an important key to the long-term risk for obesity [31], and we plan to analyze the correlation with long term health outcomes in the children of the PREOBE cohort.

Overweight and obesity are also associated with increased risk of iron deficiency [32], another well explored candidate for unfavorable perinatal programming [33]. Thus, the observed lower levels of TS, Hb, and ferritin at delivery were not unexpected but nevertheless interesting considering future neurodevelopmental assessments of the present cohort. The higher levels of ferritin at 24 weeks most likely reflect an increased inflammatory response in early pregnancy as discussed below. This and other analyses of iron metabolism in the obese group are further discussed and explored elsewhere [20], and we plan long-term follow ups to assess its impact on offspring neurodevelopment.

Our results demonstrated lower levels of vitamin B12 and folate in obese mothers compared to controls. This may partly be explained by differences in ongoing or recently discontinued intake of supplements at inclusion, but it may also reflect differences in dietary habits. Nevertheless, this observation is interesting and we found few previous assessments of vitamin B levels in obese and diabetic mothers to compare with. In one Vietnamese observational trial, authors found no association between BMI and B12 or folate [34]. Our findings are of high clinical interest since deficiencies in both vitamins are associated with maternal as well as offspring morbidity [35]. Folate deficiency is a risk factor for neural tube defects but it is also attributed a key role in fetal programming, due to its role in DNA-methylation [16]. Lower levels of vitamin B12 are associated with increased risk of neural tube defect, adiposity, increased insulin resistance, impaired neurodevelopment and altered risk of cancer in the offspring [36]. A correlation between maternal and infant B12-status was recently verified in a randomized trial from Bangladesh, suggesting that altering levels in the mother can affect the fetus [37]. Furthermore, a recent in vitro study showed that placental trophoblasts from GD mothers are less functional and sensitive to alterations in the mother and that leptin may inhibit their transport [38]. Considering these findings, the above described clinical relevance of B12, and the here observed differences in obese mothers, we hypothesize that un-optimal homeostasis of vitamin B12, may be an important mechanism behind negative long term effects following obese pregnancies. Further studies of the mechanisms for vitamin B transport in general and for mothers with obesity and/or GD may give further clues to early programming and are urgently warranted in this and other cohorts. Interestingly, even though they included overweight and obese mothers, the GD group in the present study had levels of B12 that were similar to the controls. Most likely, this is due to the local routine of offering nutritional counseling to mothers diagnosed with GD, a hypothesis supported by the higher prevalence of B12 supplementation.

Another expected maternal biochemical characteristic was the increased inflammatory response, presented through higher levels of CRP at all measures and of ESR at 24 weeks of gestation both in the overweight/obese mothers and in those with GD. This finding is in accordance with the present literature regarding obesity, which states that obesity is an inflammatory disease [39]. The clinical relevance for these small differences is unclear. However, it has been discussed that inflammation is a potential mechanism for un-optimal fetal development in obese pregnancies [40] and the results here supports further studies of this field.

The overall aim of the PREOBE project is to explore the effect of these maternal metabolic pathologies in the offspring. In the present paper, we analyzed the birth characteristics and found that the infants born from obese mothers had significantly higher birth weight and waist circumference while the infants born from mothers with GD had higher waist/height index compared to controls. Of most clinical relevance was an increased risk of macrosomia in the offspring of obese mothers. Furthermore, there were significant differences between the groups on chest circumference, showing a trend of higher levels in the obese group, even if these did not reach significance in the post hoc analyses. Altogether, the results suggest that the maternal obesity and GD contribute to small but nevertheless significant over-nutrition, further supporting the hypothesis that excess of “fuel” during pregnancy contributed to increased growth in the fetus [41]. This observation of birth weight is in line with several previous epidemiological trials [15]. We also observed a higher placental/neonatal weight ratio in the obese mothers which might suggest that placental function is compromised [42]. Other researchers have also observed increased prevalence of preterm delivery [6]. In the present paper, we found no such increased risk, possible explained by overall low prevalence (1.5–4.2 %). Neither did we observe a significant difference in infant BMI or gestational age.

A limitation with the study presented here is the heterogeneity among the mothers with GD. First, they were included in the same group independently of BMI and interactions with BMI may occur in the outcomes from this group. Secondly, the GD mothers were offered individualized nutritional recommendations and we have limited individual information regarding the compliance to this. Additional stratification of the GD mothers would have strengthened the study further but due to low sample size, we chose not to do it in the present analyses. Nevertheless, the GD group of the present study represent a common group of mothers in daily clinical practice and further knowledge regarding their metabolism during pregnancy as well as the health of their offspring, is relevant from a clinical perspective. Furthermore, since the groups were only compared to controls, the finding in the overweight and obese groups can be interpreted without a similar risk of interaction with GD. Other limitations of the study are the poor compliance rate. The dropout rate at each time of measure was high, particularly at delivery, and apart from lower power in our statistical analyses, the representativeness of the cohort may be reduced.

The main limitation of the present study is the observational design which does not allow inference of causality behind the observed differences and confounding e.g. from socioeconomic factors and normal fluctuations during pregnancy, may have occurred. However, the aims of the present baseline analyses were to identify differences and to generate candidates for future research. In that perspective, we find the results interesting and relevant. We explored our cohort using a wide variety of biomarkers and other assessments and found several interesting alterations that might hold important clues to the programming mechanisms, whether they are confounded by other factors or not. E.g., the observations in B12 levels are not described previously and prompt important future research. Above, we discussed the observations made in the present analyses, but due to the amount of data recorded, we also aim further secondary analyses to explore and discuss each group of biomarkers in detail. Furthermore, due to stored serum, placental samples, and DNA, the study enables future additional analyses. But above all, due to the planned follow ups, we are able to explore their correlation to long term health in the offspring.

## Conclusions

Maternal overweight, obesity and GD are associated with alterations in nutrition, anthropometrics, and biochemical markers of the pregnant women. The effect on the fetus is yet unclear but we observed higher body weight and increased risk of macrosomia in the offspring to mothers with obesity. Based on the alterations observed and previous research of early programming, we identified several possible candidates for future research including; markers of glucose metabolism, inflammation, and iron status, and levels of vitamin B12 and folate. Except for mechanistic studies, there is a need of long-term studies aiming to explore if any of these markers are associated with long term health in the offspring. To contribute, we plan to follow the PREOBE cohort up to 7.5 years of age.

## Abbreviations

BMI: body mass index
CRP: C-reactive protein
ESR: erythrocyte sedimentation rate
GD: gestational diabetes
Hb: hemoglobin
MCV: mean corpuscular volume
TS: transferrin saturation
TSH: thyroid-stimulating hormone
